# Predatory Behavior of *Coccinella septempunctata* on Two Different Aphid Species via Functional Response at Two Different Temperatures

**DOI:** 10.3390/biology14030245

**Published:** 2025-02-28

**Authors:** Muhammad Usama Altaf, Adeel Mukhtar, Muazzama Batool, Syed Muhammad Zaka, Rashid Azad, Yasir Hameed, Alia Tajdar, Asad Ali, Waqar Jaleel

**Affiliations:** 1 Department of Entomology, Faculty of Agricultural Sciences and Technology, Bahauddin Zakariya University, Multan 60800, Pakistan; usamaaltaf550@gmail.com (M.U.A.); adeel310545@gmail.com (A.M.); yhameed09@gmail.com (Y.H.); aliatajdar123@outlook.com (A.T.); wr_agri@yahoo.com (A.A.); 2 Cotton Research Station, Multan 60000, Pakistan; batoolmuazzama@gmail.com; 3 Department of Entomology, Faculty of Basic and Applied Sciences, The University of Haripur, Haripur 22062, Pakistan; rashidazad@uoh.edu.pk; 4 Horticultural Research Station Bahawalpur, Bahawalpur 63100, Pakistan

**Keywords:** functional response, temperature effect, *Coccinella septempunctata*, feeding potential, attack rate, handling time, biological control

## Abstract

**Simple Summary:**

*Coccinella septempunctata* (Linneaus, 1758) is a voracious predator of aphids. This study examines its functional response on two aphid species at different temperatures (15 and 25 °C) and prey densities (4, 8, 16, 32, 64, and 128 aphids). Using Roger’s random predator models and logistic regression, it was found that *C. septempunctata* adults and larvae exhibit a type II functional response. The fourth instar larvae had the highest attack rate, which increased with temperature. Higher temperatures also reduced handling times, indicating greater effectiveness in warmer climates.

**Abstract:**

*Coccinella septempunctata* (Linnaeus, 1758) is a voracious predator all over the world where aphids have found a niche. Behavioral studies of *C. septempunctata* are very important to make them effective bio-controllers. Therefore, this study explains the functional response of *C. septempunctata* praying on two distinct aphid species, examined in this work at two different temperatures. Six different prey densities (4, 8, 16, 32, 64, and 128 aphids) and two different temperatures (15 and 25 °C) were used in the experiment. All of the development stages of the predator were used to perform the experiment. Every experiment was replicated five times. The type and parameters of functional response were ascertained by the application of Roger’s random predator models and logistic regression. Results showed that *C. septempunctata* adults and larvae both had a type II functional reaction against the tested aphids in both temperatures. When comparing the fourth instar to other predatory stages, the attack rate against both aphids was shown to be greater. As the temperature rose, so did the attack rate. Fourth instar larvae exhibited attack rates of 1.314 h^−1^ on *Aphis nerii* Fonscolombe, 1758 (Hemiptera: Aphididae) and 1.959 h^−1^ on *Lipaphis erysimi* Kaltenbach, 1843 (Hemiptera: Aphididae) at 15 °C, while at 25 °C, the rates were 1.747 h^−1^ and 1.321 h^−1^, respectively. Handling time was influenced by both temperature and predator stage. As the temperature increased, the handling time of the later predatory stages decreased. This study suggests that later predatory stages of *C. septempunctatas* actively hunt aphids at higher temperatures.

## 1. Introduction

Aphids (Hemiptera: Aphididae) are the most important insect pests of agricultural, forest trees, and horticultural crops [1]. Aphids are serious pests of crops in North America [2], South America [3], Australia [4], Europe [4], Africa [5], and Asia [6]. All feeding stages of aphids cause direct and indirect damage to plants. Aphids, i.e., Oleander aphids, suck cell sap from plants [7] and secrete honeydew [8] on which sooty mold develops [9]. Moreover, aphids inject toxic saliva and transmit different viruses, such as Bean leaf virus and Pea enation mosaic virus [10]. Under unprotected conditions, *L. erysimi* can cause 80.6 to 97.6% yield losses in mustard crops [11]. Due to the continuous feeding of aphids, different losses may occur, such as a decrease in photosynthesis, stunted growth of plants, and drying of leaves [12], causing yield penalties [13]. Among aphid species, *Lipaphis erysimi* Kaltenbach, 1843 (Hemiptera: Aphididae) and *Aphis nerii* Fonscolombe, 1758 (Hemiptera: Aphididae) are serious pests of mustard crops [14].

Various controls, i.e., chemical, cultural, biological, and mechanical control methods, are being used globally to control the aphid population [13]. Among control methods, chemical control is the most prominent method used against aphids. Dimethoate and furadan are being used for the control of aphids [14]. Chemical control has several disadvantages, e.g., killing natural enemies (predators, parasitoids, etc.), creating several health problems [15], and polluting our environment [16]. Due to the continuous use of insecticides, aphids have developed resistance to several insecticides [17,18]. Biological control is one of the effective alternatives to control the aphid population instead of using highly toxic insecticides [19].

Coccinellids are known as important predators because of the wide range of hosts. These can be found in a variety of environments. Both larvae and adults have great economic importance due to their feeding nature [20]. Both stages actively feed on phytophagous soft-bodied insects, such as aphids [21], scale insects [20], thrips, whiteflies, mealybugs, and psyllids which are serious pests of agricultural ecosystems [22]. Seven spotted ladybird beetle, *Coccinella septempunctata* (Linnaeus, 1758), is an important beneficial insect because it voraciously feeds on a wide range of hosts [23]. This species has been reported in Asia [24], Europe [25], Africa [26], and North America [27].

Functional response is the rate of prey consumption by an individual predator at varying prey densities. There are three different types of functional responses, which can be seen through curves: type Ⅰ known as linear, type Ⅱ known as rectangular hyperbola, and type Ⅲ is sigmoid [28]. For the most part, invertebrate predators show type Ⅱ and tape Ⅲ functional responses [29,30]. Some important factors such as prey size [31], predator size, predator stage, experimental arena, and temperature have direct or indirect effects on the functional response [32]. Temperature is one of the major factors on the predatory potential of predators. An increase in temperature, to some extent, can increase the foraging ability of predators, but a further increase in temperature can harm the predator [33]. The effect of temperature has been investigated on different types of coccinellids [34,35]. Degree day requirements and temperature thresholds for the development may differ within species [36,37].

Temperature, as well as different prey species, both affect the functional response of the predator. Therefore, the present study was designed to determine the effect of temperature and prey species on the functional response of *C. septempunctata*.

## 2. Materials and Methods

### 2.1. Collection and Rearing

The population of the adult *C. septempunctata* was collected from wheat and canola fields located at Village No 550/T.D. A Chowk Sarwar Shaheed (30°34′34.8″ N, 71°14′03.3″ E), Muzaffargarh, Punjab, Pakistan. The population of adults was brought to the biological control laboratory, Department of Entomology, Bahauddin Zakariya University Multan. The culture of *C. septumpunctata* was reared on *L. erysimi* in the laboratory under controlled environmental conditions, i.e., 25 ± 2 °C, 60 ± 10, and 14L: 10D [30]. Plastic cages (54 × 45 × 45 cm^3^) were used for the rearing of beetles. Fifteen pairs of adults (male and female) were shifted in each plastic cage for mating and oviposition purposes. Fresh canola leaves containing *L. erysimi* were provided as the diet for beetles. Leaves containing aphids were replaced every day. Yellow cards of variable sizes were used as an oviposition substrate for *C. septumpunctata*. Moreover, *C. septumpunctata* were also used to lay eggs on the canola leaves. Eggs were collected by cutting the parts of the card and leaves having eggs and were placed in Petri dishes of 6cm diameter. After the eggs hatch, the larvae were placed separately in the Petri dishes, and *L. erysimi* were provided daily until the pupation. All four larval instars and adults of *C. septempunctata* were used in the experiment.

### 2.2. Collection of Aphid Species

Two species of aphids, i.e., *A. nerii* and *L. erysimi* were collected from their host plants, i.e., *Calotropis procera* (Aiton), and *Brassica napus* (Linnaeus), respectively, present in the Faculty of Agriculture Sciences and Technology, Bahauddin Zakariya University Multan, Punjab, Pakistan. Aphid species were identified by using a key [38].

### 2.3. Functional Response

Temperature-dependent functional response of *C. septempunctata* against two different aphid species with different densities was studied. All larval stages along with adult of *C. septempunctata* were used in this work. The feeding per beetle life stage was determined at two constant temperatures (15 and 25 °C) across six varying prey densities (4, 8, 16, 32, 64, and 128 aphids Petridish^−1 d^ day^−1^). The experiment was performed under controlled environmental conditions, 60 ± 10% RH and 14L: 10D provided in the Versatile Environmental Growth Chamber (MLR-352H, Panasonic Health Care Co., Ltd., Ora Gu, Gumna 370-0596 Japan) [39]. Adults and larvae of predators were starved for 6 h and were used singly in each replication. Five replications were performed for each treatment for five days. Counted numbers of third and fourth instars of aphids with the leaf of their host were provided as diet in a 9 cm petri dish. Following their release, they were given a one-hour period to settle and acclimate before further observation. Feeding potential was checked by counting the number of live aphids after 24 h of feeding time. Data were recorded after 24 h daily.

### 2.4. Statistical Analysis

The functional response was calculated using the statistical software R v.2.9.1 [40]. There is an integrated package in R that applies a logistic regression model [41]. The data can be distinguished into type II (negatively density-dependent) or type III (positively density dependent response) [42]. Data were arranged in MS Excel (Office 365) and then imported into R. Data were set into a FRAIR test for the results. Here is the equation for the FRAIR test [41]

$$\frac{N_{e}}{N_{{}^{\circ}}}=\frac{exp(P_{{}^{\circ}}+P_{1}N_{{}^{\circ}}+P_{2}N_{{}^{\circ}2}+P_{3}N_{{}^{\circ}3})}{1+exp(P_{{}^{\circ}}+P_{1}N_{{}^{\circ}}+P_{2}N_{{}^{\circ}2}+P_{3}N_{{}^{\circ}3})}$$

In this case, $N_{e}$ is the number of prey eaten, and $N_{{}^{\circ}}$ is initial prey density, where $P_{{}^{\circ}}$ is showing intercept $P_{1}$ is showing linear, $P_{2}$ is showing quadratic, and $P_{3}$ is showing cubic. For the estimation of these coefficients, the maximum likelihood method is used. If $P_{1}$ is greater than 0 and $P_{2}$ is smaller than 0 then it will show a type III response. If $P_{1}$ is smaller than 0 then the response to prey consumption will be negatively density-dependent, which will show a Type II response [41]. The perimeter of functional response was determined by using the Rogers II setup from the FRAIR. The equation given below was used to check different parameters of functional response [43].

$$N_{e}=N(1-e^{-a^{\prime }.p\left(Tt-N_{at.}b\right)})$$

In this formula $N_{e}$ is represents the number of prey eaten, N represents the initial density of prey, $N_{at}$ represents the prey numbers consumed by a predator, T represents the experimental time, a represents the capture rate of feeding per unit area, b is the time taken by predators to feed on the prey, and T.p is the density of attacking species. There were two different temperature responses for predatory efficiency, and the differences in these responses were compared using a paired *t*-test at a 95% confidence level using the paired *t*-test. Statistical analysis was conducted using the SAS 9.1 software package to perform this analysis.

## 3. Results

### 3.1. Functional Response of C. septempunctata against A. nerii at 15 °C

**Figure 1:**
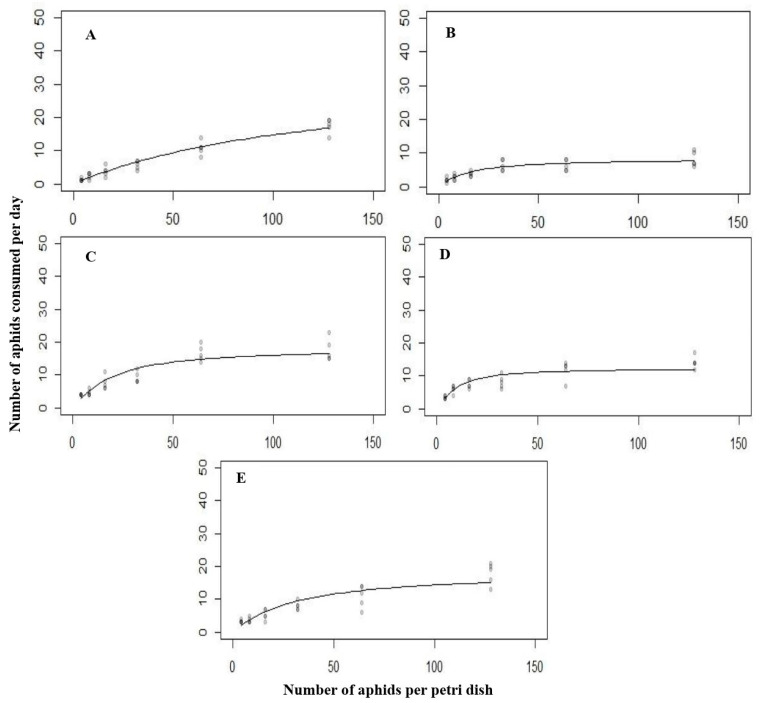
The functional response of all the predatory stages ((**A**) = first instar, (**B**) = second instar, (**C**) = third instar, (**D**) = fourth instar, (**E**) = Adult) of *C. septempunctata* against *A. nerii* at 15 °C.

The logistic regression estimates indicated a significant negative linear coefficient, as shown in Table 1. All the predatory stages of *C. septempunctata* exhibited a negative response to *A. nerii*, indicating a type Ⅱ functional response. A significant difference in the functional response was observed across the various developmental stages, i.e., first instar (Z = −3.3723, *p* < 0.0007449), second instar (Z = −7.537, *p* < 0.0001), third instar (Z = −9.847, *p* < 0.0001) fourth instar (Z = −10.507, *p* < 0.0001), and adult (Z = −6.853, *p* < 0.0001) (Figure 1).

In descending order, the attack rate against *A. nerii* was as follows: fourth instar, third instar, adult, second instar, and first instar. The extreme attack rate of 1.314 h^−1^ was observed in the fourth larval instar. Concurrently, the minimum attack rate of 0.178 h^−1^ was recorded in the first larval instar. The handling time of various developmental stages of *C. septempunctata* was as follows: second instar > fourth instar > adult > third instar > first instar (Table 2). These findings indicated that the second larval instar exhibited a maximum handling time of 0.198 h^−1^ compared to other developmental stages. At the same time, the minimum handling time of 0.052 h^−1^ was recorded in the first larval instar.

Typically, the functional response of different predatory stages of *C. septempunctata* to varying densities of *A. nerii* was of type Ⅱ. As the number of prey increased, the functional response showed a clear and rapid rise in the attack rate (Figure 1).

### 3.2. The Functional Response of C. septempunctata against L. erysimi at 15 °C

Predation of different stages of *C. septempunctata* on *L. erysimi* also exhibited a type Ⅱ functional response. The significant difference in functional response observed was Z = −7.794, *p* < 0.0001, Z = −7.955, *p* < 0.0001, Z = −12.84, *p* < 0.0001, Z = −12.938, *p* < 0.0001 and Z = −11.366, *p* < 0.0001 for all instars (fist, second, third, fourth) and adult, respectively.

The attack rate on *L. erysimi* followed this sequence: fourth instar > third instar > adult > second instar > first instar (Table 2). According to the results, the maximum attack rate of 1.747 h^−1^ was recorded in the fourth larval instar. At the same time, the minimum attack rate of 0.443 h^−1^ was recorded in the first larval instar. Similarly, handling also varied among different stages, following this order: first instar > third instar > second instar > fourth instar > adult, as indicated in Table 2. These results showed that the first instar larvae took the longest time to handle prey, which was 0.140 h^−1^. The adults took the shortest time, which was 0.0763 h^−1^.

**Figure 2:**
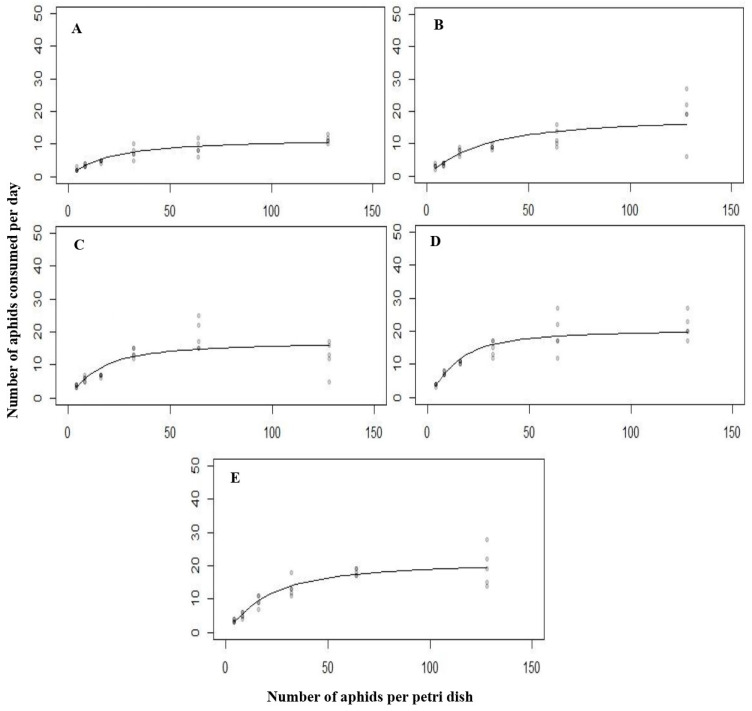
The functional response of all the predatory stages ((**A**) = first instar, (**B**) = second instar, (**C**) = third instar, (**D**) = fourth instar, (**E**) = Adult) of *C. septempunctata* against *L. erysimi* at 15 °C.

Different densities of *L. erysimi* resulting in the same type of functional response curve for *C. septempunctata* larvae and adults (Figure 2).

### 3.3. Functional Response of C. septempunctata against A. nerii at 25 °C

Logistic regression estimates showed a significant negative liner coefficient, as shown in Table 3. All predatory stages of *C. septempunctata* exhibited a negative response to *A. nerii*, characterized by a type II functional response. The functional response of the first instar (Z = −7.887, *p* < 0.0007449), second instar (Z = −11.829, *p* < 0.0001), third instar (Z = −9.596, *p* < 0.0001), fourth instar (Z = −10.232, *p* < 0.0001), and adult (Z = −10.596, *p* < 0.0001) were significant.

The attack rate on *A. nerii* was maximum for the fourth instar, followed by adult, third instar, second instar, and first instar, as shown in Table 4. The maximum attack rate of 1.960 h^−1^ was recorded in the fourth instar larvae. It was recorded in the first instar larvae, which was 1.071 h^−1^. Similarly, handling also varied among different stages of *C. septempunctata*, with the sequence being: first instar > second instar > adult > third instar > fourth instar, as shown in Table 4. According to these results, the maximum handling time was 0.0813 h^−1^ for first instar larvae. At the same time, it was recorded as the minimum for the fourth instar larvae, which was 0.0170 h^−1^.

**Figure 3:**
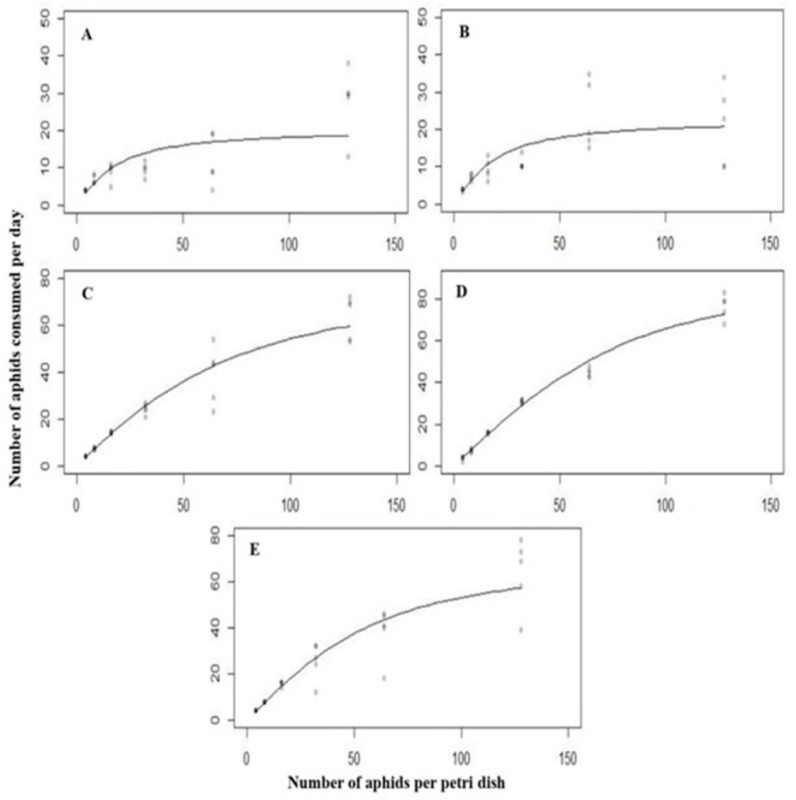
The functional response of all the predatory stages ((**A**) = first instar, (**B**) = second instar, (**C**) = third instar, (**D**) = fourth instar, (**E**) = Adult) of *C. septempunctata* against *A. nerii* at 25 ° C.

In response to different densities of *A. nerii* and *C. septempunctata* all predatory stages exhibited a typical type II functional response. The attack rate increased rapidly in this function as the number of prey increased (Figure 3).

### 3.4. The Functional Response of C. septempunctata against L. erysimi at 25 °C

Predation of different stages of *C. septempunctata* on *L. erysimi* also showed type Ⅱ functional responses. The functional response of the first instar (Z = −13.277, *p* < 0.0001), second instar (Z = −9.451, *p* < 0.0001), third instar (Z = −11.577, *p* < 0.0001), fourth instar (Z = −3.022, *p* < 0.002508), and adult (Z = −10.323, *p* < 0.0001) were significant. The attack rate on *L. erysimi* was recorded at a maximum for the third instar larvae, followed by the adult, fourth instar, second instar, and second instar, as shown in Table 4. According to the results, the third instar larvae exhibited a maximum attack of 1.927 h^−1^. At the same time, it was recorded as the minimum for second instar larvae, which was 1.012 h^−1^. Similarly, the handling time also varied among various stages of *C. septempunctata*, with maximum handling time recorded in the first instar larvae followed by the second instar, third instar, adult, and fourth instar, as shown in Table 4.

**Figure 4:**
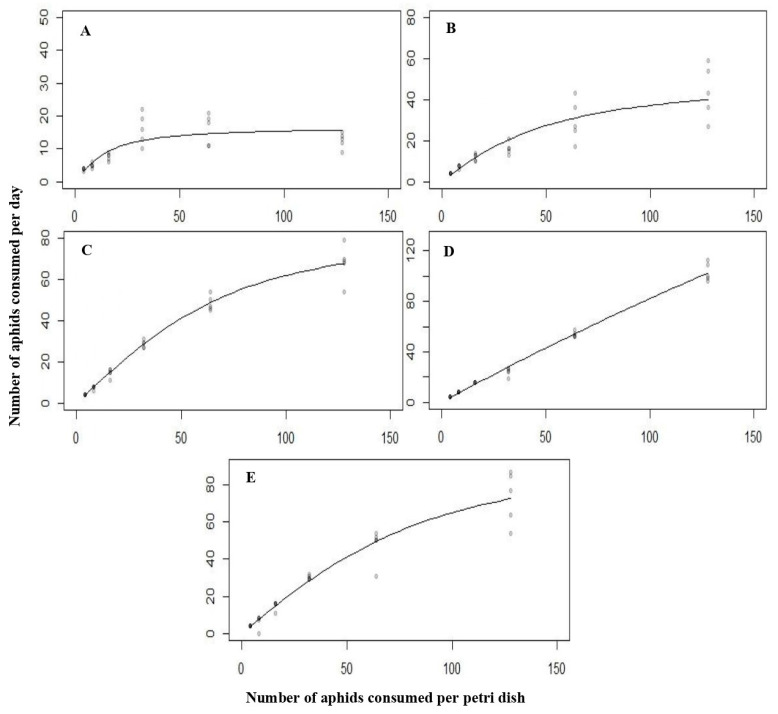
The functional response of all the predatory stages ((**A**) = first instar, (**B**) = second instar, (**C**) = third instar, (**D**) = fourth instar, (**E**) = Adult) of *C. septempunctata* against *L. erysimi* at 25 °C.

When different densities of *L. erysimi* were given as diet, the same type of functional response curves were obtained for the different larval instars and adults of *C. septempunctata* (Figure 4). It was concluded that the later predatory stages of *C. septempunctata* have proven to be the most effective predators of *A. nerii* and *L. erysimi*. Additionally, temperature had a notable effect on the functional responses and attack rates of *C. septempunctata* when preying on *A. nerii* and *L. erysimi* (Table 5 and Table 6).

## 4. Discussion

For *C. septempunctata*, a linear correlation coefficient was found for the predatory life stage. It is concluded from our results that temperature and hosts have a significant effect on the functional response of *C. septumpuncata*. Both predatory stages (larvae and adult) of *C. septempunctata* showed a Type Ⅱ functional response against the different densities of *A. nerii* and *L. erysimi* at different temperatures. This study represents the fitness of *C. septempunctata* on two different species of aphid. The *C. septumpuncata* have multiple prey hosts, and this quality of predator is most important because the fitness maintained by different nutrients only one prey cannot fulfill the requirement of a predator [4,44]. Such type of response was also recorded on different insect species by different scientists under different conditions, i.e., *Oenopia conglobate* Linneaus, 1758 (Coleoptera: Coccinellidae) praying on *Agonoscena pistaciae* Burckhardt and Lauterer, 1989 (Hemiptera: Aphalaridae) at different temperatures [45], *Harmonia axyridis* Pallas, 1773 (Coleoptera: Coccinellidae) feeding on *Acyrthosiphon pisum* Harris, 1776 (Hemiptera: Aphididae) at various temperatures [46], *Harmonia axyridis* and *C. septempunctata* feeding on *Aphis glycines* Matsumura, 1917 (Hemiptera: Aphididae) [47], *H. axyridis* praying on *Cacopsylla chinensis* Yang and Li, 1981 (Hemiptera: Psyllidae) [48], *C. septempunctata* feeding on *L.erysimi* [49], *C. carnea*, *C. septempunctata*, and *Hippodamia variegate* Goeze, 1777 (Coleoptera: Coccinellidae) praying on changing numbers of aphids [50], *Cycloneda sanguinea* Linneaus, 1763 (Coleoptera: Coccinellidae) and *Scymnus levaillanti* Mulsant, 1850 (Coleoptera: Coccinellidae) feeding on *Aphis gossypii* Glover, 1877 (Hemiptera: Aphididae) [51]. There is, however, a difference in the response of predators at different stages. As *Eriopis connexa* Germar, 1824 (Coleoptera: Coccinellidae) adults exhibited a type III functional response when feeding on *Maxrosiphum euohoribae* Thomas, 1878 (Hemiptera: Aphididae) [52] (Table 7).

Important factors that describe the amount of functional response are the attack rate and handling time. The attack rate is the speed at which a predator can bring its prey to hand in a specific length of time, while handling time is the amount of time spent looking for each host. In our findings, when exposed to different densities of two aphids (*A. nerii* and *L. erysimi*) at different temperatures, the attack rate and handling time differed for all predatory stages. We found that predator feeding capacity increases as prey density increases. Feeding capacity decreases with decreasing temperature. As shown by the estimates of attack rate, the fourth and third instar larvae of *C. septempunctata* are the most effective predatory stages against both tested aphid species at 15 and 25 °C, respectively. Results revealed that the attack rate of early instars on both aphid species was relatively low compared to later ones. Similar findings were also reported on other coccinellid predators, i.e., the 4th instar of *H. variegata* and *Adalia tetraspilota* showed relatively higher attack rates when fed *A. pomi*, *A. craccivora*, and *B. brassicae* [53], likewise higher attack rates were exhibited by the 4th instar of *Oenopia conglobata* by feeding on *Agonoscena pistaciae* at different temperatures [45]. Our results indicated that the attack of the predatory stages is higher at high temperatures and lower at low temperatures. A temperature-dependent study of *H. axyridis* praying on *Acyrthosiphon pisum* showed that the attack rate is high at higher temperatures compared to lower temperatures, which is similar to our findings [46]. Based on our results, the handling time for the later predatory stages was maximum at 15 °C and minimum at 25 °C. Feeding of *C. septempunctata* on *M. persicae* at different temperatures also supports our findings that later predatory stages have maximum handling time at lower temperatures compared to higher temperatures [33]. Analyzing consumption as a fraction of body weight can offer a more nuanced understanding of the feeding ecology of different instars. For instance, earlier instars might have lower absolute attack rates due to their smaller size, but if their consumption per unit body weight is comparable to or higher than later instars, it would suggest they are equally or more efficient relative to their size. Conversely, if later instars have higher relative consumption rates, it could emphasize the increasing metabolic demands and predatory efficiency with maturation.

The current investigation showed that the attack rate of early predatory stages is low, and handling time is high, which shows that early predatory stages are not effective predators. However, the later predatory stages have the highest attack rate and lowest handling time. The conclusion is that the later stages of predatory organisms are competent predators. Similar to our results, the fourth instar of *C. septempunctata* proved to be the best predatory stage praying on *A. pisum* by exhibiting the highest attack rate and lowest handling time [54]. Similarly, later predatory stages of *C. septempunctata* praying on *L. erysimi* and *B. brassicae* showed the lowest handling time as well as the highest attack rate [55]. The coccinellids beetles (*C. septempunctata*, *H. variegata*) and *C. carnea* feeding on *A. craccivora* and *M. persicae* also showed that the later predatory stages have the lowest handling time as well as the highest attack rate [50]. Likewise, another research work supporting our results, i.e., early stages of *H. dimidiata* praying on *A. gossypii* showed low attack rates and higher handling time, but later stages exhibited the lowest handling time as well as the highest attack rate [56].

According to recent research, temperature has an impact on *C. septumpuncata’s* functional response when it feeds on various aphid species. At 15 °C and 25 °C, there is a noticeable variation in the attack rate and handling time. The attack rate is low, and the handling time is long at 15 °C, while the opposite is true at 25 °C, where the handling time is short, and the attack rate is high. Our findings are supported by the observation that, at lower temperatures than at other high temperatures, handling times are longer, and the attack rate of later predatory stages is lower when *H. axyridis* prays on *A. pisum* [46]. The feeding of *O. conglobata* on *A. pistaciae* at different temperatures showed that at higher temperatures the attack rate is high and handling time is low compared to lower temperatures [45].

## 5. Conclusions

In conclusion, the later predatory stages of *C. septempunctata* are the most effective predators of *A. nerii* and *L. erysimi*, performing best at 25 °C. They are highly suitable for biological control programs in greenhouses and fields. Looking ahead, rising global temperatures may enhance their efficacy, suggesting that future pest management strategies could leverage these climatic changes for improved results. In future work can also be done on genetic adaptation and its integration into sustainable pest management.

## Figures and Tables

**Table 1:** The proportion of prey of *A. nerii* and *L. erysimi* by the various stages of *C. septempunctata* at 15 °C using maximum likelihood logistic regression.

Aphid Species	Predatory Stages	Estimate	S.E *	z Value	Pr (>z)
*Aphis nerii*	1st Instar	−0.006	0.001	−3.373	7.449 × 10^−4^ **
	2nd Instar	−0.015	0.002	−7.537	4.797 × 10^−14^ **
	3rd Instar	−0.015	0.002	−9.847	<2.2 × 10^−16^ **
	4th Instar	−0.018	0.002	−10.507	<2.2 × 10^−16^ **
	Adult	−0.011	0.002	−6.853	7.216 × 10^−12^ **
*Lipaphis erysimi*	1st Instar	−0.014	0.002	−7.794	6.483 × 10^−15^ **
	2nd Instar	−0.012	0.002	−7.955	1.791 × 10^−15^ **
	3rd Instar	−0.021	0.002	−12.84	<2.2 × 10^−16^ **
	4th Instar	−0.020	0.002	−12.938	<2.2 × 10^−16^ **
	Adult	−0.017	0.002	−11.366	<2.2 × 10^−16^ **

* S.E. stands for standard error; ** Results are highly significant (*p* < 0.001).

**Table 2:** Coefficient of attack rate (a) and handling time (T_h_) of *C. septempunctata* from Rogers Ⅱ model using *A. nerii* and *L. erysimi* as prey (T= 1 day) at 15 °C.

Aphid Species	Insect Stage		Attack Rate		Handling Time		
		*a*	±S.E *	Pr (>|z|)	Ta (h)	±S.E *	Pr (>|z|)
*Aphis nerii*	1st Instar	0.178	0.030	2.792× 10^−9^ **	0.052	0.015	4.207 × 10^−4^ **
	2nd Instar	0.409	0.111	0.000232 **	0.198	0.028	8.562 × 10^−13^ **
	3rd Instar	0.803	0.154	1.995× 10^−7^ **	0.091	0.009	<2.2 × 10^−16^ **
	4th Instar	1.314	0.316	3.197× 10^−5^ **	0.133	0.012	<2.2 × 10^−16^ **
	Adult	0.438	0.087	4.253× 10^−7^ **	0.092	0.0128	6.774 × 10^−13^ **
*Lipaphis erysimi*	1st Instar	0.443	0.100	9.494× 10^−6^ **	0.140	0.018	2.163 × 10^−14^ **
	2nd Instar	0.545	0.100	5.326× 10^−8^ **	0.089	0.010	<2.2 × 10^−16^ **
	3rd Instar	1.082	0.194	2.475× 10^−8^ **	0.096	0.008	<2.2 × 10^−16^ **
	4th Instar	1.747	0.312	2.144× 10^−8^ **	0.080	0.006	<2.2 × 10^−16^ **
	Adult	0.950	0.149	2.031× 10^−8^ **	0.076	0.007	<2.2 × 10^−16^ **

* S.E. stands for standard error; ** Represents data are statistically (*p* < 0.001).

**Table 3:** The proportion of prey *A. nerii* and *L. erysimi* by the various stages of *C. septempunctata* at 25 °C using maximum likelihood logistic regression.

Aphid Species	Predatory Stages	Estimate	S.E *	z Value	Pr (>z)
*Aphis nerii*	1st Instar	−0.011	0.001	−7.886	3.104 × 10^−15^ ***
	2nd Instar	−0.018	0.001	−11.829	<2.2 × 10^−16^ ***
	3rd Instar	−0.013	0.001	−9.596	<2.2 × 10^−16^ ***
	4th Instar	−0.017	0.001	−10.232	<2.2 × 10^−16^ ***
	Adult	−0.015	0.001	−10.596	<2.2 × 10^−16^ ***
*Lipaphis erysimi*	1st Instar	−0.023	0.002	−13.277	<2.2 × 10^−16^ ***
	2nd Instar	−0.013	0.001	−9.451	<2.2 × 10^−16^ ***
	3rd Instar	−0.019	0.002	−11.577	<2.2 × 10^−16^ ***
	4th Instar	−0.005	0.002	−3.022	2.508 × 10^−3^ **
	Adult	−0.017	0.002	−10.323	<2.2 × 10^−16^ ***

* S.E. stands for standard error; *** Results are highly significant (*p* < 0.001) ** Results are significant (*p* < 0.01).

**Table 4:** Attack rate (a) and handling time (Th) coefficients for *C. septempunctata* from the Rogers Ⅱ model at 25 °C, *A. nerii* and *L. erysimi* as prey (T = 1 day).

Aphid Species	Insect Stage		Attack Rate		Handling Time		
		*A*	±S.E *	Pr (>|z|)	T_h_ (h)	±S.E *	Pr (>|z|)
*Aphis nerii*	1st Instar	1.071	0.241	8.627 × 10^−6^ **	0.081	0.009	<2.2 × 10^−16^ **
	2nd Instar	1.214	0.216	1.883 × 10^−8^ **	0.074	0.007	<2.2 × 10^−16^ **
	3rd Instar	1.371	0.132	<2.2 × 10^−16^ **	0.020	0.002	<2.2 × 10^−16^ **
	4th Instar	1.959	0.185	<2.2 × 10^−16^ **	0.017	0.001	<2.2 × 10^−16^ **
	Adult	1.718	0.185	<2.2 × 10^−16^ **	0.023	0.001	<2.2 × 10^−16^ **
*Lipaphis erysimi*	1st Instar	1.155	0.198	6.404 × 10^−9^ **	0.098	0.008	<2.2 × 10^−16^ **
	2nd Instar	1.012	0.120	<2.2 × 10^−16^ **	0.032	0.003	<2.2 × 10^−16^ **
	3rd Instar	1.927	0.172	<2.2 × 10^−16^ **	0.019	0.001	<2.2 × 10^−16^ **
	4th Instar	1.321	0.102	<2.2 × 10^−16^ **	0.004	0.001	4.344 × 10^−4^ **
	Adult	1.746	0.150	<2.2 × 10^−16^ **	0.016	0.001	<2.2 × 10^−16^ **

* S.E. stands for standard error; ** Represents data are statistically (*p* < 0.001).

**Table 5:** Comparative mean (±S.E) feeding data at different temperatures (15 °C and 25 °C) at different predatory stages of *C. septempunctata* on *A. nerii*.

No. of Aphids Per Treatment	First Larval Instar		Second Larval Instar		Third Larval Instar		Fourth Larval Instar		Adult	
	15 °C	25 °C	15 °C	25 °C	15 °C	25 °C	15 °C	25 °C	15 °C	25 °C
4	1.23 ± 0.21	3.95 ± 0.05 *	2.10 ± 0.25	3.84 ± 0.12 *	3.80 ± 0.09	3.95 ± 0.05 *	3.27 ± 0.17	3.52 ± 0.39 *	3.12 ± 0.21	4.00 ± 0.00 *
8	2.41 ± 0.45	6.70 ± 0.54 *	2.84 ± 0.22	7.03 ± 0.39 *	4.53 ± 0.30	7.72 ± 0.17 *	5.96 ± 0.50	7.11 ± 0.37 *	3.52 ± 0.43	8.00 ± 0.00 *
16	3.73 ± 0.65	9.07 ± 1.08 *	3.76 ± 0.27	9.25 ± 1.15 *	7.56 ± 0.94	14.13 ± 0.36 *	7.72 ± 0.55	15.80 ± 0.20 *	5.44 ± 0.70	15.52 ± 0.39 *
32	5.74 ± 0.63	9.62 ± 0.79 *	6.48 ± 0.63	10.65 ± 0.91 *	9.31 ± 0.74	24.13 ± 0.99 *	8.06 ± 0.89	31.33 ± 0.29 *	8.12 ± 0.62	22.84 ± 5.90 *
64	10.76 ± 0.96	12.00 ± 3.06 *	8.24 ± 1.05	23.72 ± 4.19 *	16.28 ± 1.12	38.53 ± 5.57 *	11.68 ± 1.23	45.02 ± 0.96 *	11.16 ± 1.57	38.04 ± 5.13 *
128	17.12 ± 0.97	28.07 ± 4.09 *	9.44 ± 1.17	21.00 ± 4.83 *	17.68 ± 1.50	63.64 ± 4.21 *	14.40 ± 0.79	76.44 ± 2.60 *	17.80 ± 1.37	63.56 ± 6.95 *

* Feeding at both temperatures was significantly different at particular densities and predatory stages by paired *t*-test (*p* = 0.05).

**Table 6:** Comparative mean (±S.E) feeding data at different temperatures (15 °C and 25 °C) at different predatory stages of *C. septempunctata* on *L. erysimi*.

No. of Aphids Per Treatment	First Larval Instar		Second Larval Instar		Third Larval Instar		Fourth Larval Instar		Adult	
	15 °C	25 °C	15 °C	25 °C	15 °C	25 °C	15 °C	25 °C	15 °C	25 °C
4	2.16 ± 0.17	3.60 ± 0.19 *	3.08 ± 0.29	4.00 ± 0.00 *	3.64 ± 0.12	4.00 ± 0.00 *	3.76 ± 0.12	3.96 ± 0.04 *	3.36 ± 0.25	4.00 ± 0.00 *
8	3.38 ± 0.30	4.80 ± 0.41 *	3.56 ± 0.07	7.50 ± 0.39 *	5.96 ± 0.44	7.60 ± 0.40 *	7.48 ± 0.10	8.00 ± 0.00 *	5.4 ± 0.33	6.08 ± 1.54 *
16	4.52 ± 0.19	7.50 ± 0.50 *	7.41 ± 0.44	11.80 ± 0.87 *	6.8 ± 0.17	14.62 ± 0.99 *	11.84 ± 1.41	12.83 ± 0.64 *	9.44 ± 0.76	14.92 ± 0.98 *
32	7.21 ± 0.85	15.90 ± 2.19 *	8.72 ± 0.36	15.90 ± 1.39 *	13.84 ± 0.58	28.50 ± 0.74 *	15.04 ± 0.97	24.16 ± 1.44 *	13.2 ± 1.17	30.32 ± 0.55 *
64	8.76 ± 1.02	15.93 ± 2.20 *	11.96 ± 1.30	29.30 ± 4.48 *	18.76 ± 1.91	48.30 ± 1.66 *	19.00 ± 2.39	53.52 ± 0.92 *	18.24 ± 0.50	47.44 ± 4.28 *
128	11.48 ± 0.48	12.20 ± 1.07 *	18.56 ± 3.49	43.70 ± 5.90 *	12.48 ± 2.09	68.13 ± 3.96 *	21.44 ± 1.79	103.10± 3.23 *	19.73 ± 2.53	73.52 ± 6.28 *

* Feeding at both temperatures was significantly different by paired *t*-test (*p* = 0.05).

**Table 7:** Summary of different types of functional responses exhibited by different biocontrol agents against different pests.

Name of Biocontrol Agent	Target Pest	Functional Response Type	References
*Oenopia conglobate*	*Agonoscena pistaciae*	Type II	[45]
*Harmonia axyridis*	*Acyrthosiphon pisum*	Type II	[46]
*Harmonia axyridis*	*Aphis glycines*	Type II	[47]
*Coccinella septempunctata*	*Aphis glycines*	Type II	[47]
*Harmonia axyridis*	*Cacopsylla chinensis*	Type II	[48]
*Coccinella septempunctata*	*L.erysimi*	Type II	[49]
*Chrysoperla carnea*	*Aphid* sp.	Type II	[50]
*Coccinella septempunctata*	*Aphid* sp.	Type II	[50]
*Hippodamia variegata*	*Aphid* sp.	Type II	[50]
*Cycloneda sanguinea*	*Aphis gossypii*	Type II	[51]
*Scymnus levaillanti*	*Aphis gossypii*	Type II	[51]
*Eriopis connexa*	*Maxrosiphum euohoribae*	Type III	[52]

## Data Availability

The data will be available on request.
